# Clinical Evaluation of PolyDeep, A Computer-Aided Detection System: A Multicenter Randomized Tandem Colonoscopy Trial

**DOI:** 10.3390/diagnostics15212751

**Published:** 2025-10-30

**Authors:** Pedro Davila-Piñón, Astrid Irene Díez Martín, Alba Nogueira-Rodríguez, Ruben Domínguez-Carbajales, Florentino Fdez-Riverola, Sara Zarraquiños, Luisa de Castro, Jesús Herrero, Nereida Fernández, Pablo Vega, David Remedios, Alfonso Martínez, Manuel Puga, Sara Alonso, Noel Pin, Natalia García-Morales, Laura Rivas, Alejandro Ledo, Ramiro Macenlle, Lucia Cid, Antonio Rodríguez, Santiago Soto, Franco Baiocchi, Indhira Pérez-Medrano, Eloy Sánchez, Daniel Glez-Peña, Miguel Reboiro-Jato, Hugo López-Fernández, Joaquín Cubiella

**Affiliations:** 1 Research Group in Gastrointestinal Oncology Ourense, University Hospital of Ourense, 32005 Ourense, Spain; 2 Fundación Pública Galega de Investigación Biomédica Galicia Sur, Hospital Universitario de Ourense, Servicio Gallego de Salud (SERGAS), 32005 Ourense, Spain; 3 Department of Computer Science, ESEI—Escuela Superior de Ingeniería Informática, Universidade de Vigo, 32004 Ourense, Spainriverola@uvigo.gal (F.F.-R.); hlfernandez@uvigo.gal (H.L.-F.); 4 SING Research Group, Galicia Sur Health Research Institute (IIS Galicia Sur), Servicio Gallego de Salud (SERGAS)-Universidad de Vigo (UVIGO), 32004 Ourense, Spain; 5 Department of IT, University Hospital of Ourense, 32005 Ourense, Spain; 6 Department of Gastroenterology, University Hospital of Ourense, 32005 Ourense, Spainpablo.vega.villaamil@sergas.es (P.V.);; 7 Department of Gastroenterology, University Hospital Álvaro Cunqueiro of Vigo, 36312 Vigo, Spain; 8 Department of Gastroenterology, Complexo Hospitalario Universitario de Pontevedra, 36071 Pontevedra, Spain; 9 Instituto de Investigación Sanitaria Galicia Sur (IISGS), 36312 Vigo, Spain

**Keywords:** colorectal cancer, screening, adenoma miss rate, tandem colonoscopy, polyp detection, CADe, colonoscopy quality, surveillance, positive FIT, advanced polyps

## Abstract

**Background/Objectives**: Computer-aided detection (CADe) systems are increasingly used in endoscopy to enhance lesion recognition. PolyDeep is a CADe/x tool previously assessed in an observational study. The aim of our study is to determine if PolyDeep-assisted colonoscopy reduces the adenoma miss rate (AMR) compared with conventional colonoscopy. **Methods**: We carried out a multicenter randomized controlled trial with a tandem colonoscopy design in participants from a colorectal cancer screening program (positive fecal immunochemical test-FIT or surveillance). Expert endoscopists performed all colonoscopies, and patients were allocated to groups by a computer-generated sequence. The primary endpoint was AMR; secondary endpoints included polyp miss rate (PMR), serrated lesion miss rate (SLMR) and advanced polyp miss rate (APMR). **Results**: From May to November 2023, we recruited 260 patients and excluded 20, leaving 240 for analysis. Baseline characteristics were balanced between groups (62.1% male; mean age 62.3 ± 6.5 years; 65.8% FIT-positive; mean first withdrawal time 13:38 ± 08:07 min; mean second withdrawal time 07:50 ± 03:38 min; lesion detection rate 76.6%; mean polyps per patient 3.4 ± 3.1). We did not find statistically significant differences between PolyDeep-assisted and conventional colonoscopy groups in AMR (21.3% vs. 18.1%, *p* = 0.5), PMR (21.8% vs. 20.3%, *p* = 0.7), SLMR (23.4% vs. 25.6%, *p* = 0.9) or APMR (7.3% vs. 11.3%, *p* = 0.5). In the subgroup analysis according to indication, we did not find any statistically significant differences. **Conclusions**: In the context of a CRC screening program, PolyDeep-assisted colonoscopy did not reduce AMR.

## 1. Introduction

Colorectal cancer (CRC) is the third most commonly diagnosed malignancy and the second leading cause of cancer-related mortality worldwide, with the highest incidence and mortality rates observed globally [1]. CRC typically develops over a number of years from precancerous lesions, such as adenomas and serrated polyps [2]. Organised CRC screening programmes are offered to individuals at average risk (generally those aged between 50 and 75 years) reducing both the incidence and mortality of the disease [2,3,4]. The primary screening modalities employed to detect precancerous lesions are the faecal immunochemical occult blood test (FIT) and colonoscopy [3,5]. In several countries, including Spain, FIT is the initial test offered; individuals with a positive result are subsequently referred for diagnostic colonoscopy, which remains the gold standard for lesion detection [3,5].

Colonoscopy remains the only test capable of detecting precancerous lesions, namely adenomas and serrated lesions, within the colonic mucosa [3,5]. A proportion of these lesions present challenges owing to their small size (i.e., <5 mm) or morphology (e.g., flat or depressed), which can hinder their detection [6,7]. In addition, factors related to endoscopist performance such as fatigue, suboptimal mucosal exposure, or limited experience may further compromise lesion identification [6,7]. Collectively, these factors contribute to the risk of missed lesions during colonoscopy, which may subsequently lead to the development of post-colonoscopy colorectal cancer (PCCRC) [4]. The adenoma miss rate (AMR), defined as the proportion of adenomas detected during a second withdrawal relative to the total number of adenomas, ranges from 17% to 48%, with two meta-analyses estimating average miss rates of 22% and 26%, respectively [8,9].

In recent years, various computer-aided diagnosis (CAD) systems based on artificial intelligence (AI) and deep learning (DL) have been applied to medical image analysis, including radiography, mammography, and cardiovascular imaging [10,11,12]. In the context of colonoscopy, computer-aided detection (CADe) systems have been evaluated in randomized clinical trials employing a range of designs (i.e., comparisons between CADe-assisted and conventional colonoscopy, or tandem colonoscopy designs) and using diverse endpoints, including adenoma detection rate (ADR), polyp detection rate (PDR), AMR, and polyp miss rate (PMR) [13,14,15,16,17,18,19,20,21,22]. Although the use of these systems appears to improve diagnostic performance metrics, their role in routine clinical practice remains to be fully established [23]. The comprehensive clinical validation of PolyDeep was designed to evaluate whether the system achieved a performance comparable to commercially available CADe solutions and to explore its feasibility for market implementation.

PolyDeep is a computer-aided detection and characterization (CADe/x) system developed to identify colorectal lesions in real time during colonoscopy. The system integrates a neural detection network based on the YOLOv3 architecture and an object tracking algorithm, which maintains and follows all detections in each subsequent frame [24,25,26,27]. PolyDeep was initially validated in vitro and subsequently evaluated in a prospective study using a second-observer design [28,29]. In the present randomized controlled trial with a tandem colonoscopy design, we aimed to determine whether the AMR (i.e., primary endpoint) in the PolyDeep-assisted colonoscopy group is superior to that of the conventional colonoscopy group.

## 2. Materials and Methods

### 2.1. Study Design

PolyDeep Advance 2 (NCT05512793) is a multicentre randomised controlled trial (RCT) with a tandem colonoscopy design. The study was conducted in the endoscopy units of three university hospitals in Spain: Hospital de Ourense, Hospital Álvaro Cunqueiro de Vigo, and Hospital de Montecelo de Pontevedra. The study was approved by the Institutional Review Board of Pontevedra-Vigo-Ourense (protocol code: 2022/067 and date of approval: 17 February 2022) in accordance with the Declaration of Helsinki and applicable guidelines for good clinical practice. Data from the three centers were verified and monitored within the electronic Case Report Form (eCRF) in the REDCap platform at the Galicia-Sur health research institute (https://redcap.tic1-iisgaliciasur.es/; accessed on 17 November 2023). The study was reported in accordance with the CONSORT-AI guideline for randomized trials.

### 2.2. Participants of the Study

We prospectively included patients aged between 40 and 79 years who underwent a colonoscopy after a positive FIT or a surveillance colonoscopy following the resection of advanced adenomas. Participants were excluded if they had a personal history of colorectal cancer or previous colonic resection, a hereditary syndrome predisposing to colorectal cancer, or serrated polyposis syndrome. Regarding colonoscopy quality, patients were excluded if they had an inadequate Boston Bowel Preparation Scale score (<2 in any segment or <6 overall) or if cecal intubation was not achieved. All participants provided written informed consent prior to inclusion in the study.

### 2.3. Randomization Process

We randomized the patients in a 1:1 ratio by a stratified block design based on the colonoscopy indication, such as positive FIT or surveillance endoscopy. We created blocks of two in all possible combinations (i.e., four combinations) and randomly assigned each block to positions within the randomization template. To minimize bias, we doubled the number of allocation slots (i.e., with 260 patients to be randomized, we generated 520 slots), ensuring a more balanced and unbiased randomization process. Finally, the randomization template was uploaded to the eCRF by the study coordinator. The endoscopists performed the randomization process of the participants before the colonoscopy, to know which is the allocation group and what tandem colonoscopy they should do. However, participants were not aware of the randomization allocation.

### 2.4. Clinical Setting

We conducted the study in a conventional endoscopy room using a standard endoscopy tower with high definition colonoscopes using the model EXERA III CV 190 or higher. We integrated the PolyDeep system into the setup, connecting the CADe/x system to both the endoscopy tower and the monitor displaying the colonoscopy image. The same monitor showed both the colonoscopy image and the PolyDeep system’s output, including overlays highlighting detected polyps as previously described. Upon the patient’s arrival in the endoscopy room, the endoscopists explained the study and obtained written informed consent. They then randomized the participants in the eCRF, assigning them to either the conventional group by performing conventional colonoscopy and then assisted colonoscopy, or PolyDeep group performing an assisted colonoscopy first and after that a conventional colonoscopy. The 20 endoscopists participating in the study were experts, meaning they are participants of the CRC screening program with more than 300 colonoscopies, one year of experience and with a rigorous quality control based on key quality indicators. Each endoscopist recruited a uniform number of participants during the clinical trial. They performed a back-to-back colonoscopy with two withdrawal phases according to the assigned randomization sequence. During each withdrawal phase, the endoscopists measured withdrawal time using the timer in the colonoscope control device. Lesions were counted only in one of the withdrawal phases and resected upon identification. Throughout the procedure, the nursing and auxiliary team recorded polyp data on a log sheet, including withdrawal phase, morphology, location, size, and both the endoscopists’ and PolyDeep’s optical diagnosis.

We sent all identified and retrieved lesions for histopathological evaluation, which served as the gold standard for analysis. If a lesion could not be resected during the procedure, the endoscopists documented its characteristics and obtained a biopsy. In the eCRF, we recorded participant demographic data (age, sex, and colonoscopy indication), colonoscopy details (Boston Bowel Preparation Scale score, withdrawal time, and cecal intubation), and polyp-specific information (withdrawal phase of identification, morphology, size, location, optical diagnosis, and histological evaluation)

### 2.5. Endpoints

The primary endpoint was to assess and compare the AMR between the conventional group and the PolyDeep group. Secondary endpoints were the evaluation and comparison of the PMR, serrated lesion miss rate (SLMR), advanced adenoma miss rate (AAMR), advanced serrated lesion miss rate (ASLMR) and advanced polyp miss rate (APMR) between both groups.

### 2.6. Sample Size

We calculated the sample size based on the assumption of an AMR of 30% in the conventional colonoscopy group and 20% in the PolyDeep group, with a two-sided alpha level of 0.05 and a beta error of 0.20 (0.80 power) [14]. Based on an average of 2.5 lesions detected per colonoscopy, this corresponds to approximately 118 patients and 294 adenomas per arm. Attending to a 10% drop-out rate due to incomplete colonoscopies, the final sample size was estimated at 260 patients.

### 2.7. Statistical Analysis

We conducted a descriptive analysis of the study population, including demographics, colonoscopy procedures, and identified lesions. Quantitative variables are reported as mean and standard deviation, while qualitative variables expressed as frequency and percentages. We compared both groups using the t-Student and chi-square tests. To evaluate and compare miss rates of different types of lesions, we built 2 × 2 confusion matrices. For the primary endpoint, we calculated the AMR as the ratio between the number of adenomas detected during the second withdrawal and the total number of adenomas detected in the colonoscopy (i.e., first and second withdrawals). For secondary endpoints, we determined the PMR, SLMR, AAMR, ASLMR and APMR using similar calculations, dividing the number of lesions detected during the second withdrawal by the total number of detected lesions. Additionally, we performed subgroup analyses based on location, lesion size and indication. All miss rate metrics are presented as percentages, with a significant difference between both groups established in *p* < 0.05 for the t-Student and chi-square tests. We performed all statistical analyses using R (version 4.4.1, The R Foundation for Statistical Computing, Institute for Statistics and Mathematics, Vienna, Austria).

## 3. Results

### 3.1. Population Description

**Figure 1:**
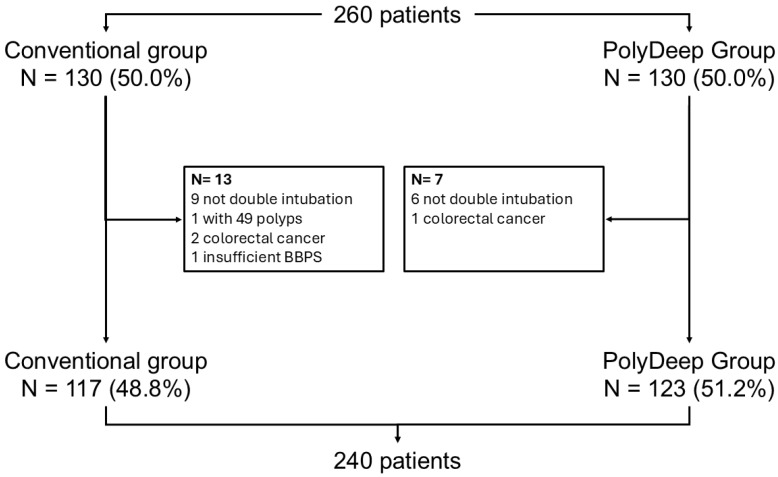
Flowchart of the study.

Between May and November 2023, we randomly allocated 260 patients, with 130 (50%) assigned to each group. After excluding 20 patients (13 from the conventional group and 7 from the PolyDeep group), we analysed data from 240 participants, with 48.8% in the conventional group and 51.2% in the PolyDeep group (Figure 1). Table 1 presents a description and comparison of the baseline characteristics of both groups. Most participants included by the endoscopists were male and had a positive FIT result. We did not find statistically significant differences between the conventional group and the PolyDeep group (Table 1).

We detected 674 lesions of which 612 (90.8%) were adenomas and serrated lesions, while 62 (9.2%) belonged to other categories (i.e., inflammatory polyps, harmatomatous polyps or lesions without histology). These lesions were distributed as 445 adenomas (66.0%), 167 serrated lesions (24.8%), 37 other polyps (5.2%) and 27 lesions without histology (4.0%) (Table 2). Table 2 shows the overall number of lesions detected in each group.

Related to the characteristics of the lesions identified, most of them were diminutive polyps located in the proximal colon with more detections in this category made by the conventional group than by the PolyDeep group (Table 2). The mean size of polyps in both groups was inferior to 5 mm (i.e., conventional group: 4.5 ± 4.7 vs. PolyDeep group: 4.9 ± 4.7). The mean number of adenomas detected per patient in the conventional group was 2.9 ± 2.5, while in the PolyDeep group 2.8 ± 2.2.

### 3.2. Diagnostic Performance: Adenoma Miss Rate, Polyp Miss Rate, Serrated Lesion Miss Rate

Table 2 shows the distribution of lesions detected in the first and in the second withdrawal to determine the lesion miss rates. We did not find statistically significant differences for AMR between the conventional group and the PolyDeep group (18.1% vs. 21.3%, *p* = 0.5). Similarly, we did not find statistically significant differences between both groups for PMR (20.3% vs. 21.8%, *p* = 0.7) and SLMR (25.6% vs. 23.4%, *p* = 0.9).

### 3.3. Sub-Analysis by Size, Location and Advanced Lesions

In the size-based analysis, we did not find statistically significant differences in the miss rates of diminutive polyps smaller than 5 mm (24.1% vs. 25.5%, *p* = 0.8), in small polyps with less than 10 mm (22.8% vs. 23.4%, *p* = 0.9), and large polyps which are equal or larger than 5 mm (11.4% vs. 15.2%, *p* = 0.6) (Table 2).

With respect to the polyp miss rates by location (i.e., proximal and distal colon; Table 2) we did not observe statistically significant differences between conventional and PolyDeep groups (proximal colon: 18.5% vs. 19.3%, *p* = 0.8; distal colon: 22.8% vs. 24.7%, *p* = 0.8). Finally, there were no significant differences between both groups in advanced polyps (11.3% vs. 7.3%, *p* = 0.5), advanced adenomas (4.8% vs. 5.1%, *p* = 1.0) and advanced serrated lesions (35.7% vs. 13.6%, *p* = 0.2).

### 3.4. Sub-Analysis by Colonoscopy Indication

Table 3 shows the distribution of lesions detected by screening indication. In this case, we did not find statistically significant differences for AMR (14.9% vs. 20.4%, *p* = 0.2) between the conventional and PolyDeep groups. We also did not find statistically significant differences for SLMR (29.4% vs. 25.0%, *p* = 0.7) and PMR (18.6% vs. 21.6%, *p* = 0.5). For the miss rates by advanced lesions, location, and size, we did not find statistically significant differences between both groups. On the other hand, for surveillance colonoscopy, we did not find significant differences between both groups for AMR, SLMR, and PMR. As well as we did not find differences for advanced lesions (33.3% vs. 0.0%, *p* = 0.2), location (proximal: 18.8% vs. 26.3%, *p* = 0.5 distal: 34.4% vs. 17.2%, *p* = 0.2), and size (<5 mm: 28.2% vs. 26.9%, *p* = 1.0; ≥5 mm: 0.0% vs. 6.7%, *p* = 0.5).

## 4. Discussion

In this RCT with a tandem colonoscopy design, the use of PolyDeep did not reduce the AMR in the context of screening colonoscopies performed by expert endoscopists. Moreover, no significant differences were observed between groups with respect to indication, lesion type, or lesion location. In recent years, a substantial number of CADe systems have been integrated into real-time colonoscopy procedures, consistently demonstrating reductions in the AMR and increases in the ADR [13,18,20,30]. The adoption of CADe systems appears to enhance colonoscopy quality reflected by key performance indicators, such as ADR.

Our study employed a robust design, utilizing a back-to-back (i.e., tandem colonoscopy) approach with the AMR as the primary endpoint. One of the strengths of this design is that tandem colonoscopy allows for reliable AMR assessment with a relatively small sample size. However, this design also presents several potential limitations. First, AMR is not a standard quality indicator routinely measured in clinical practice, which may limit the generalizability of our findings. Second, there is a potential for bias among endoscopists due to the awareness of a second withdrawal opportunity; if a lesion was missed during the first withdrawal, they had a second chance to detect it, possibly altering their performance. Additionally, endoscopists fatigue may have affected outcomes, as double colonoscopies were performed within a standard clinical workload. This was particularly relevant towards the end of the day, when cumulative fatigue may have increased the likelihood of missed lesions. Furthermore, the use of a CADe system such as PolyDeep may have introduced an unintended sense of competition with the technology, potentially leading endoscopists to detect fewer lesions when the system was active. Different study designs were used to evaluate AMR. In our study, the same expert endoscopist performed a classical back-to-back colonoscopy (i.e., first withdrawal conventional and second assisted, or vice versa). However, in another study assessing the same outcome, patients were randomized to undergo either assisted colonoscopy with CAD-EYE or non-assisted colonoscopy. In that study, trainee endoscopists performed the first withdrawal, and expert endoscopists performed the second withdrawal to assess AMR. In contrast to our findings, that study reported a lower AMR in the CAD-EYE–assisted group compared with the conventional group [17].

As a limitation, the subgroup analysis by indication (i.e., screening or surveillance colonoscopies) reported interesting results, but the sample size calculations were based on the primary endpoint (i.e., AMR). This is the reason why the relevance of these findings was limited, as was not the main focus of our clinical trial and the required sample size would have been different. We found two additional limitations related to polyp size and endoscopist training. First, most of the lesions identified in our study were diminutive polyps, which may have negatively affected PolyDeep’s performance in detecting lesions of this type. It is important to note that diminutive polyps predominate in both study arms. Second, endoscopists did not perform any trial colonoscopies or receive training with PolyDeep prior to the start of the study. This lack of familiarization may have temporarily influenced their performance during the initial procedures until they became familiar to the PolyDeep interface.

A metanalysis of four tandem colonoscopy studies reported a 65% reduction in AMR (odds ratio 0.35, 95% CI: 0.25–0.49), along with a 78% reduction in the SLMR [13]. Another metanalysis reported comparable reductions in PMR and AMR, with absolute risk differences of 19% and 17.5%, respectively [18]. Further, recent evidence supports these findings, showing a 55% reduction in SLMR with CADe-assisted colonoscopy without reaching statistical significance [30]. However, our study did not identify statistically significant differences in AMR between PolyDeep-assisted and conventional colonoscopy. A non-significant reduction in SLMR was observed, consistent with previous meta-analyses. Additionally, the difference in withdrawal time was minimal, with the CADe-assisted colonoscopy in our study taking only seven seconds longer, comparable to findings from another study, which reported a nine-second increase [30].

One possible limitation of our study that may explain our negative results is the high expertise of the endoscopists who participated in the trial. As an example, the AMR of the conventional group was clearly inferior to the AMR reported in literature [14]. In this sense, in a recently published RCT, CADe did not increase the advanced adenoma detection rate in the context of FIT-based CRC screening programs [31]. In fact, the screening endoscopists involved in our study underwent periodic evaluation using the ADR (i.e., most of the endoscopists had an ADR superior to the 60%) as a measure of colonoscopy quality [32]. The high ADR of our endoscopists may partly explain why a 10% difference in AMR between assisted and conventional colonoscopy was not achieved. Furthermore, the visual distraction caused by the bounding boxes displayed on the monitor could have influenced performance during the first assisted withdrawal. As a matter of fact, the latest guideline from the European Society of Gastrointestinal Endoscopy reported only a weak recommendation for the routine use of CADe, due to limited supporting evidence and ongoing concerns regarding its implementation, particularly the need for further studies on cost-effectiveness and the potential drawbacks of human-AI interaction [23].

PolyDeep integrates a YOLOv3 neural detection network with an object tracking algorithm, trained on polyp detection with polyp and not-polyp images [24,25,26,28]. A potential limitation that could affect detection performance may be the algorithm itself. At the start of this clinical trial, a more recent version of the YOLO algorithm (i.e., YOLOv8) was already available, offering an improved lesion detection performance [33]. In an ex vivo study, YOLOv8 demonstrated high diagnostic performance for polyp detection in polyp images, with a sensitivity of 91.7% and an F1 score of 92.4% [34]. However, no clinical validation or RCTs have yet evaluated YOLOv8 in real-time colonoscopy procedures to determine whether it would outperform PolyDeep in detection tasks. Furthermore, as the clinical validation of PolyDeep was already underway, modifying the neural network architecture was not appropriate, as it would have compromised comparability with the results of the preceding observational detection study [29].

The results obtained in our study are consistent with the latest guidelines published, which provide a weak recommendation for the use of CADe in routine colonoscopy. These guidelines indicate that CADe systems have not yet demonstrated a meaningful difference in key colonoscopy quality outcomes, such as ADR or CRC incidence [23]. In the future, we plan to evaluate PolyDeep’s impact on ADR in a multicenter randomized clinical trial (NCT05513261) to determine whether assisted colonoscopy can improve this key quality indicator. Additionally, PolyDeep could be evaluated in two further studies: one aimed at assessing its potential to enhance the diagnostic performance of trainee or non-experienced endoscopists in polyp detection, and another to investigate the influence of PolyDeep’s visual signals on endoscopists’ decision-making process.

To conclude, the first PolyDeep-assisted colonoscopy did not reduce the AMR compared to the first conventional colonoscopy, indicating that endoscopists missed a relatively small number of lesions. Similarly, in the subgroup analyses by indication and polyp type, no statistically significant differences were observed between both groups.

## Figures and Tables

**Table 1:** Comparison between colonoscopy group and PolyDeep group.

	Conventional Group ^1^ (N = 117)	PolyDeep Group ^2^ (N = 123)	p ^3^
**Age (years) ^4^**	63.0 ± 6.8	61.6 ± 6.2	0.1
**Sex (male) ^4^**	69 (59.0%)	80 (65.0%)	0.4
**Indication (FIT)**	75 (64.1%)	83 (67.5%)	0.7
**Boston Bowel cleansing**	7.59 ± 1.28	7.42 ± 1.31	0.3
**First withdrawal time (minutes: seconds)**	13.34 ± 8.39	13.41 ± 07.37	0.9
**Second withdrawal time (minutes: seconds)**	7.58 ± 3.17	7.42 ± 3.57	0.6
**Detection of lesions (yes)**	90 (76.9%)	94 (76.4%)	0.7
**Number of polyps**	3.4 ± 3.3	3.4 ± 2.9	1.0
**Polyp size (millimetres)**	4.5 ± 4.7	4.9 ± 4.7	0.4

^1^ First withdrawal with conventional colonoscopy; ^2^ first withdrawal with PolyDeep (i.e., assisted colonoscopy); ^3^ comparisons between groups with the chi-square and t-Student test with a significant level of *p* < 0.05; ^4^ categorical variables are presented as frequency and percentage and continuous variables as mean and standard deviation.

**Table 2:** Number of lesions detected in the first and second withdrawal.

	Conventional Group ^1^		PolyDeep Group ^2^		
	1st Withdrawal	2nd Withdrawal ^3^	1st Withdrawal	2nd Withdrawal ^3^	p ^4^
**Adenoma**	172 (81.9%)	38 **(18.1%)**	185 (78.7%)	50 **(21.3%)**	0.5
**Polyp ^5^**	239 (79.7%)	61 **(20.3%)**	244 (78.2%)	68 **(21.8%)**	0.7
**Serrated lesion**	67 (74.4%)	23 **(25.6%)**	59 (76.6%)	18 **(23.4%)**	0.9
**Other polyp**	12 (75.0%)	4 (25.0%)	16 (84.2%)	3 (15.8%)	-
**Not histology**	12 (66.7%)	6 (33.3%)	6 (66.6%)	3 (33.3%)	-
**Advanced adenoma ^6^**	40 (95.2%)	2 **(4.8%)**	37 (94.9%)	2 **(5.1%)**	1.0
**Advanced serrated lesion ^7^**	9 (64.3%)	5 **(35.7%)**	19 (86.4%)	3 **(13.6%)**	0.2
**Advanced polyp ^8^**	47 (88.7%)	6 **(11.3%)**	51 (92.7%)	4 **(7.3%)**	0.5
**Proximal polyp ^9^**	141 (81.5%)	32 **(18.5%)**	134 (80.7%)	32 **(19.3%)**	0.8
**Distal polyp ^10^**	98 (77.2%)	29 **(22.8%)**	110 (75.3%)	36 **(24.7%)**	0.8
**<5 mm polyp**	161 (75.9%)	51 **(24.1%)**	149 (74.5%)	51 **(25.5%)**	0.8
**<10 mm polyp**	203 (77.2%)	60 **(22.8%)**	209 (76.6%)	64 **(23.4%)**	0.9
**≥5 mm polyp**	78 (88.6%)	10 **(11.4%)**	95 (84.8%)	17 **(15.2%)**	0.6

^1^ First conventional colonoscopy; ^2^ first PolyDeep-assisted colonoscopy; ^3^ variables are described as frequency and percentages; ^4^ chi-square test with a level of significance *p* < 0.05; ^5^ polyp include adenomas and serrated lesions; ^6^ adenomas with >10 mm, tubule-villous or villous histology and high grade of dysplasia; ^7^ serrated lesions with >10 mm or dysplasia; ^8^ include advanced adenomas and advanced serrated lesions; ^9^ polyp between cecum and splenic flexure; ^10^ polyp between descendent and rectum.

**Table 3:** Miss rates of lesions by indications.

	Screening ^1^		p ^4^	Surveillance ^1^		p ^4^
	Conventional Group ^2^	PolyDeep Group ^3^		Conventional Group ^2^	PolyDeep Group ^3^	
**Adenoma miss rate**	$\frac{22}{148}$ (14.9%) ^5^	$\frac{37}{181}$ (20.4%)	0.2	$\frac{16}{62}$ (25.8%)	$\frac{13}{54}$ (24.1%)	1.0
**Polyp miss rate ^6^**	$\frac{37}{199}$ (18.6%)	$\frac{53}{245}$ (21.6%)	0.5	$\frac{24}{101}$ (23.8%)	$\frac{15}{67}$ (22.4%)	1.0
**Serrated lesion miss rate**	$\frac{15}{51}$ (29.4%)	$\frac{16}{64}$ (25.0%)	0.7	$\frac{8}{39}$ (20.5%)	$\frac{2}{13}$ (15.4%)	1.0
**Advanced polyp miss rate ^7^**	$\frac{3}{44}$ (6.8%)	$\frac{4}{49}$ (8.2%)	1.0	$\frac{3}{9}$ (33.3%)	$\frac{0}{6}$ (0.0%)	0.2
**Proximal polyp miss rate ^8^**	$\frac{19}{104}$ (18.3%)	$\frac{22}{128}$ (17.2%)	0.9	$\frac{13}{69}$ (18.8%)	$\frac{10}{38}$ (26.3%)	0.5
**Distal polyp miss rate ^9^**	$\frac{18}{95}$ (18.9%)	$\frac{31}{117}$ (26.5%)	0.3	$\frac{11}{32}$ (34.4%)	$\frac{5}{29}$ (17.2%)	0.2
**<5 mm polyp miss rate**	$\frac{27}{127}$ (21.3%)	$\frac{37}{148}$ (25.0%)	0.6	$\frac{24}{85}$ (28.2%)	$\frac{14}{52}$ (26.9%)	1.0
**≥5 mm polyp miss rate**	$\frac{10}{72}$ (13.9%)	$\frac{16}{97}$ (16.5%)	0.8	$\frac{0}{16}$ (0.0%)	$\frac{1}{15}$ (6.7%)	0.5

^1^ Screening colonoscopy after faecal immunochemical occult blood test or surveillance after resection of colorectal adenomas; ^2^ first conventional colonoscopy; ^3^ first PolyDeep-assisted colonoscopy; ^4^ chi-square test with a level of significance *p* < 0.05; ^5^ variables are presented as the ratio between the lesions missed and the total number of that type of lesion and percentage.; ^6^ polyps include adenomas and serrated lesions; ^7^ advanced lesion miss rate include adenomas >10 mm, tubule-villous or villous histology and high-grade dysplasia. For serrated lesions > 10 mm and dysplasia; ^8^ polyps between cecum and splenic flexure; ^9^ polyps between descendent and rectum.

## Data Availability

The data supporting the findings of this study are available from the corresponding author upon reasonable request.
